# New Integrated Model Approach to Understand the Factors That Drive Electronic Health Record Portal Adoption: Cross-Sectional National Survey

**DOI:** 10.2196/11032

**Published:** 2018-11-19

**Authors:** Jorge Tavares, Tiago Oliveira

**Affiliations:** 1 NOVA Information Management School Universidade NOVA de Lisboa Lisboa Portugal

**Keywords:** electronic health records, adoption, eHealth, patients, patient portals

## Abstract

**Background:**

The future of health care delivery is becoming more patient-focused, and electronic health record (EHR) portals are gaining more attention from worldwide governments that consider this technology as a valuable asset for the future sustainability of the national health care systems. Overall, this makes the adoption of EHR portals an important field to study.

**Objective:**

The aim of this study is to understand the factors that drive individuals to adopt EHR portals.

**Methods:**

We applied a new adoption model that combines 3 different theories, namely, extended unified theory of acceptance and use of technology, health belief model, and the diffusion of innovation; all the 3 theories provided relevant contributions for the understanding of EHR portals. To test the research model, we used the partial least squares causal modeling approach. We executed a national survey based on randomly generated mobile phone numbers. We collected 139 questionnaires.

**Results:**

Performance expectancy (beta=.203; *t*=2.699), compatibility (beta=.530; *t*=6.189), and habit (beta=.251; *t*=2.660) have a statistically significant impact on behavior intention (*R*^2^=76.0%). Habit (beta=.378; *t*=3.821), self-perception (beta=.233; *t*=2.971), and behavior intention (beta=.263; *t*=2.379) have a statistically significant impact on use behavior (*R*^2^=61.8%). In addition, behavior intention (beta=.747; *t*=10.737) has a statistically significant impact on intention to recommend (*R*^2^=69.0%), results demonstrability (beta=.403; *t*=2.888) and compatibility (beta=.337; *t*=2.243) have a statistically significant impact on effort expectancy (*R*^2^=48.3%), and compatibility (beta=.594; *t*=6.141) has a statistically significant impact on performance expectancy (*R*^2^=42.7%).

**Conclusions:**

Our research model yields very good results, with relevant *R*^2^ in the most important dependent variables that help explain the adoption of EHR portals, behavior intention, and use behavior.

## Introduction

### Overview

The electronic health record (EHR) portal or an EHR patient portal is a technology that combines an EHR system and a patient portal where patients can communicate with their health care providers (eg, send messages, schedule medical appointments, and request prescription refills online) and access their EHR and medical exams results [1-3]. EHR is a repository of patient data in a digital form, stored and exchanged securely. EHRs may include a range of data such as medical history, medication and allergies, immunization status, laboratory test results, radiology images, vital signs, personal statistics like age and weight, and billing information [4]. EHR portals have received great attention at the governmental level worldwide [2,3,5]. In the United States, the support given to EHRs, via a meaningful use program, led the federal government to commit unparalleled resources to support the adoption of EHRs through incentive payments that can reach up to US $27 billion over 10 years [5,6]. EHR portals are a relevant topic not only in the United States but also in Europe through several projects such as the European Patients Smart Open Services (EpSOS) initiative promoted by the European Union Commission [3]. EpSOS focuses on developing a practical information and communication technology infrastructure that will enable secure access to patient information, including EHR among different European countries [3].

Understanding the adoption and use of EHR portals by patients is a very relevant topic with clear benefits for the society and future sustainability of the different health care systems in the world [4,7]. The warning signs are that the number of patients with chronic diseases is projected to grow by 45% between 2007 and 2025, and the health care providers’ workforce will be 10% smaller [8]. Combining these 2 trends, there will be less health care professionals available in the future to provide support to the patients. EHR portals may help patients carry out self-management activities, making the use of the health care system more effective and sustainable, not only from the patient care standpoint but also from the financial perspective due to the increasing cost of the health care budget in different countries [3,4,8-10]. Regarding EHR portal users’ sociodemographic characteristics, there is a consistent trend to be younger and more educated than the population average [11-13].

Most of the EHR portals’ usage in the developing countries ranges between 5% and 10% of the total annual target population that they aim to reach [3,14]. Most of the EHR portals are implemented at an organizational or health care unit level, but there are some examples of EHR portals that have been implemented at the national level [3,14]. Probably the most successful nationwide implementation of an EHR portal is the Sundhed portal in Denmark with 1.1 million unique registered users, approximately 20% coverage of the Danish population [14]. In Portugal, both public and private health care institutions have EHR portals [3]. The most relevant public EHR portal is the National Health Service (NHS) portal that in its first implementation was not very successful, but in its new release, which was launched recently, provides a higher level of security (2-factor authentication) and broader access to the patients to their clinical information across NHS [3,7]. Recently in Portugal, we also have good examples of investment by private health care groups, like the EHR portal MyCuf [7]. What we also perceive in the private health care institutions such as MyCuf is the exclusive delivery of the medical examination results and other documentation online, increasing the use of this online platform and making it compulsory, something that is not yet a policy in the NHS [3,7]. Due to the fact that some of the private health care providers perceived an efficiency advantage, they have invested in developing more sophisticated portals than the public hospitals, but the new version of the NHS portal has now also started aggregating all the patient information from the different public health care institutions, making it available to the patient at one place [7]. There are differences between the different types of EHR portals in the same country and among different countries, but the most common and frequently used features identified in the literature, which generally apply to an EHR portal, are as follows: management of health information and communication with health providers, medical appointments schedule, check their own EHR, and request for medical prescription renewals [2-4,15-17]. Taking into account the relevance of all the current initiatives that are ongoing in Portugal regarding EHR portals, a nationwide survey using a sample of randomly generated mobile numbers was applied in our study.

The goals of this study are to estimate the percentage of EHR portal users among the Portuguese population and understand the factors that drive health care consumers to adopt and use EHR portals. We apply 3 different theories to build our research model: the extended unified theory of acceptance and use of technology (UTAUT2), the health belief model (HBM) theory, and the diffusion of innovation (DOI) theory. In the Research Model section, a more detailed rationale explaining why we combined these 3 theories is provided.

### Theoretical Background

The goal of our study is to focus on the adoption of the EHR portals from the standpoint of the health care consumer. According to the literature, assessing the adoption of eHealth tools by health care consumers still demands more effort due to the persisting low number of studies published to date and in view of the importance of the topic [3,4]. The most frequently used adoption models when studying eHealth adoption by health care professionals are the unified theory of acceptance and use of technology [2,18,19] and the technology acceptance model (TAM) [3,20,21]. When evaluating the studies published in the field of consumer health information technology adoption, most of the research studies use TAM or extensions of TAM [22-25]. Although the studies that used extended TAM used other models and theories with TAM to adapt it to the consumer health technology context (see Table 1), TAM was not envisaged with consumer focus in mind. Rather, we need a model developed for the consumer use setting, and UTAUT2 was developed precisely with this purpose, achieving good results [26]. A recent study using a UTAUT2 extension demonstrated its usefulness in assessing the critical determinants for the adoption of EHR portals in which the construct habit, which is a consumer-specific construct, was the one with the greatest impact on the adoption of EHR portals [2]. This fact shows the importance of using research models that are consumer specific.

**Table 1:** eHealth patient-focused adoption models.

Theory	Dependent variable	Findings	Reference
TAM^a^, integrated model, motivational model	eHealth behavioral intention	Users’ perceived technology usefulness, users’ perceived ease of use, intrinsic motivation, and extrinsic motivation have significant positive impact on behavioral intention Integrated model does not have better results than TAM or motivational model when predicting behavioral intention	[25]
UTAUT2^b^ plus CFIP^c^ (cross-country analysis: United States vs Portugal)	Behavioral intention and use behavior in EHR^d^ portals	Behavioral intention drivers are performance expectancy, effort expectancy, social influence, hedonic motivation, price value, and habit. The predictors of use behavior are habit and behavioral intention Social influence, hedonic motivation, and price value are only predictors in the US group Confidentiality issues do not seem to influence acceptance	[27]
TAM, Trust and Privacy	Intention to adopt eHealth	Perceived ease of use, perceived technology usefulness, and trust are significant predictors	[22]
UTAUT2	Behavioral intention and use behavior in EHR portals	The behavioral intention drivers are performance expectancy, effort expectancy, social influence, and habit Habit and behavioral intention are drivers of use behavior	[7]
DOI^e^ (mix of qualitative and quantitative study)	Adoption rate of an e-appointment scheduling service	The influence of the perceived attributes of the e-appointment scheduling service according to the DOI theory helps explaining the low adoption and use Low socioeconomic status and lower educational level negatively influence the e-appointment scheduling service adoption rate	[13]
Extended TAM in health information technology	Health information technology behavioral intention	Perceived ease of use, perceived technology usefulness, and perceived threat significantly influenced health consumer behavioral intention	[23]
UTAUT2 extended model	Behavioral intention and use behavior in EHR portals	Effort expectancy, performance expectancy, habit, and self-perception are predictors of behavioral intention Habit and behavioral intention are predictors of use behavior	[2]
Institutional theory and UTAUT^f^	Patient portal use behavior	Coercive and mimetic pressures significantly influence patient portal use behavior Normative pressure was found to be not relevant	[28]

^a^TAM: technology adoption model.

^b^UTAUT2: extended unified theory of adoption and use of technology.

^c^CFIP: concern for information privacy.

^d^EHR: electronic health record.

^e^DOI: diffusion of innovation.

^f^UTAUT: unified theory of adoption and use of technology.

Although EHR portals are consumer-oriented technologies, because a patient can be viewed as a health care consumer, the use of a model like UTAUT2 should not be regarded sufficient to explain the complexity of EHR portal adoption [2,23,26]. Several studies that used constructs or frameworks related to the HBM demonstrated their usefulness and statistical significance in explaining health information consumer adoption [2,23,29]. The HBM advocates that belief in health risk predicts the likelihood of engaging in health behavior, or an alternative way to look into it is to consider that the perceived severity, instead of the real severity, of the health complaint could be the driving force behind the action [23,30]. Evidence in the literature shows that the global usage of EHR portals is still limited [2,5,14,31].

As the rate of adoption is still low in the use of EHR portals, literature that has addressed the eHealth patient technologies under the scope of DOI also mentioned a low level of global use and identified the users as early adopters [13,32]. Earlier studies that focused on understanding eHealth patient-centered technologies and EHR portals identified both performance expectancy and effort expectancy as important predictors of behavioral intention to use [2,7,23,25]. Both performance expectancy and effort expectancy have their equivalents within DOI theory as relative advantage and complexity [32,33], providing another strong argument to use DOI theory when studying EHR portals [7,13]. This study included intention to recommend as a dependent variable. According to our knowledge, this is the first time that intention to recommend is studied in the field of the adoption of EHR portals [2,22,23,27,33]. Understanding whether current users of new technologies that have a low level of adoption can be used to promote them is a valuable asset that should be evaluated [33].

### Research Model

**Figure 1:**
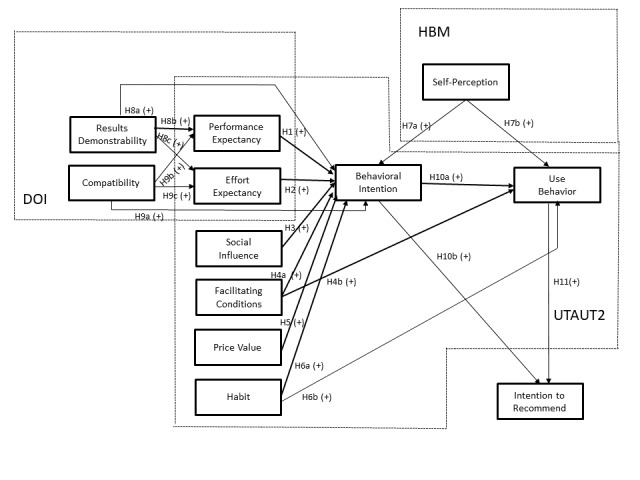
The research model. DOI: diffusion of innovation; HBM: health belief model; UTAUT2: extended unified theory of acceptance and use of technology.

As EHR portals are a new technology focused on consumer health [2,3], our research model is a combination of UTAUT2 [26], self-perception, a construct from the HBM [2,23,30,34,35], and a framework based on the DOI model [32,33,36]. On the basis of the extensive literature review and previous studies, the need to have a model with patient-centric focus was identified, something that UTAUT2 provides, with consumer-specific constructs and good results in previous eHealth and EHR portal studies [2,14]. As we are studying the EHR portals from the heath care consumers’ perspective and not from the health care providers’ standpoint, it is relevant to use UTAUT2, because most of the existing (information technology) IT adoption models are not consumer-specific [2,15]. In addition, previous research identified the relevance of including health care–specific constructs in studying the adoption of EHR portals and eHealth platforms [3,16,17]; therefore, we used a construct related to HBM that already achieved good results within the scope of our study [3]. EHR portals are a new eHealth technology and the DOI model revealed in past studies to be able to explain the adoption of new eHealth tools successfully, therefore making it suitable to be used to study EHR portals adoption [13,18]. We also made some improvements in our research model concerning the theories we used. In the UTAUT2 framework, we did not use the construct hedonic motivation. Hedonic motivation is conceptualized as intrinsic motivation (eg, pleasure or enjoyment) [26]. People use EHR portals frequently when they are ill [1] and that can be viewed by many as not being an enjoyable activity [37]. Recent literature confirms no consistent and relevant results in predicting the adoption of EHR portals with hedonic motivation [2,7,27]. Literature evidence shows that constructs related to the HBM, such as perceived health risk or self-perception, are much better motivation predictors of adoption of EHR portals than hedonic motivation [2,23]. We also used intention to recommend as a dependent variable. This is a variable that has not been used in the literature to explain the adoption of EHR portals [2,27,38]. Instead, it has been used in other technologies to explain adoptions such as mobile payments [33], which were also regarded as relatively new and with a low usage level [33] like EHR portals [2]. In these types of technologies, providers start to rely on current or potential users to recommend them to others [33]. That is why we included intention to recommend in our research model. Figure 1 illustrates the new research model.

### Extended Unified Theory of Acceptance and Use of Technology Constructs

Performance expectancy is theorized to be the degree to which using a specific technology provides benefits to consumers in executing particular tasks [26,39]. Overall, patients adopt and use more eHealth tools and EHR portals that provide benefits in executing online health-related activities [2,7,23,25]:

H1: Performance expectancy will positively influence behavioral intention.

Effort expectancy is the degree of ease connected to consumers’ usage of a certain technology [26,39]. The simpler it is for health care consumers to use an EHR portal, the greater is the likelihood that they will use it [2,7,23,25]:

H2: Effort expectancy will positively influence behavioral intention.

Social influence is the extent to which people acknowledge that others who are significant to them believe they should use a particular technology [2,23,25]. According to the literature, social influence plays a role in the adoption of eHealth and EHR portals, because patients with the same health issues tend to be induced by others sharing the same or similar condition [27,28,40,41]:

H3: Social influence will positively influence behavioral intention.

Facilitating conditions refers to consumers’ awareness of the support and resources available to execute a particular behavior [26,39]. A possible barrier to patients’ use of eHealth tools is the nonexistence of resources or support services that enable them to access and use these types of technology, implying that health care consumers with better conditions favor EHR portals usage and adoption [26,27,42]:

H4(a): Facilitating conditions will positively influence behavioral intention.

H4(b): Facilitating conditions will positively influence use behavior.

If we relate to the consumer environment, price value is a relevant dimension, because consumers usually bear the costs linked with purchasing products and services [26]. If health care consumers can obtain the results of their medical examination online, for example, through an EHR portal, they save time and transportation costs by avoiding an unnecessary trip to a clinic or hospital [27,43]:

H5: Price value will positively influence behavioral intention.

Habit can be described as the degree to which people tend to perform behaviors automatically due to learning [26]. According to recent literature, habit positively influences the use and adoption of eHealth tools and EHR portals [27,44]:

H6(a): Habit will positively influence behavioral intention.

H6(b): Habit will positively influence use behavior.

The role of behavioral intention has been recognized in eHealth with the literature affirming that the driver of use and adoption of EHR portals is preceded by the behavioral intention to use them [2,23,25,27]:

H10(a): Behavioral intention will positively influence use behavior.

### Health Behavior Construct

Supporting the concept of self-perception is the HBM. HBM assumes that subjective health concerns determine whether individuals execute a health-related action such as making an appointment with their physician [30]. Self-perception in health [30,34,35] posits that the perceived (rather than the real) severity of the health complaint could be the driving force inducing the action [30,35,45].

There is evidence in the literature that self-perception influences behavior intention to use eHealth tools and EHR portals [2,23]:

H7(a): Self-perception will positively influence behavioral intention.

There is also evidence in the literature that self-perception can not only drive intentions but also directly influence actions with regard to the use of health-related services [2,23,30]. Often with sensitive topics and particularly with health-related topics, a mismatch between intentions and effective actions may occur [4,27,46]. It is then also relevant to evaluate the potential positive effect of self-perception on use behavior:

H7(b): Self-perception will positively influence use behavior.

### Diffusion of Innovation Constructs

Roger’s DOI theory is one of the most acknowledged theories for studying IT adoption [13]. According to DOI, innovation is an idea, technology, or a process that is perceived as unknown or new to a particular group of individuals [13,47]. Diffusion is how the information about the innovation is shared inside the social system [47]. The attributes of an innovation comprise 5 user-perceived qualities: relative advantage, compatibility, complexity, trialability, and observability [47]. Moore and Benbasat [36] expanded the original set of innovation attributes proposed by DOI to be applicable to the IT setting. One example was the construct observability, which was subdivided into results demonstrability and visibility [36]. Subsequent studies have found that results demonstrability is more relevant than visibility in predicting users’ intention to use a technology, particularly in IT health care [32]. We did not measure trialability because there was no evidence that our target population has participated in a trial usage of EHR portals [3]. EHR portals should be seen as a new technology that relates to the concept of an innovation in consumer IT within the scope of health care.

Relative advantage is the extent to which the consumer perceives improvements or benefits upon the current technology by adopting an innovation [47]. Relative advantage measures fundamentally the same thing as performance expectancy within the context of DOI [32,33]. Complexity measures the extent to which an innovation is difficult to understand or be used [47]. We also find a commonality between effort expectancy and complexity [32,33]. Both relative advantage and complexity within the context of DOI, according to the literature, may be regarded as positively influencing the behavioral intention to adopt EHR portals [2,13,27,32].

Results demonstrability is the degree to which the tangible results of adopting and using an innovation can be visible and then communicable [36]. According to the literature, this may have a direct effect on the behavioral intention to use an EHR portal [13,32]. In addition, potential users can better comprehend the benefits of using a new eHealth technology when noticeable results of the tool are directly evident, advocating a positive connection between results demonstrability and performance expectancy [32]. The degree to which a specific individual noticed the results of using an innovation to be demonstrable partially reflects belief in using the tool and more easily achieving the desired outcome [13,32]. Thus, we theorize and ground on the literature that results demonstrability will positively influence effort expectancy:

H8(a): Results demonstrability will positively influence behavioral intention.

H8(b): Results demonstrability will positively influence performance expectancy.

H8(c): Results demonstrability will positively influence effort expectancy.

Compatibility measures the extent to which an innovation is perceived as being aligned with the existing consumer lifestyle values and current and past experiences [47]. Compatibility has demonstrated to be a predictor of the behavioral intention to adopt a new technology in general, and also in consumer eHealth [13,33]. Compatibility, also like results demonstrability, is an antecedent of performance expectancy and effort expectancy [13,33]. Users may perceive EHR portals to be more compatible if they see advantages in using them to manage specific health care activities without additional complexity [2,13,33]. Compatibility consequently strengthens performance expectancy, effort expectancy, and behavioral intention to use EHR portals [2,13,33]:

H9(a): Compatibility will positively influence behavioral intention.

H9(b): Compatibility will positively influence performance expectancy.

H9(c): Compatibility will positively influence effort expectancy.

### Users’ Intention to Recommend Electronic Health Record Portals

IT consumers with a greater intention to adopt a new technology are more likely to become users and to recommend that specific technology to others [33,48]. Often with sensitive topics and particularly with health-related topics, a mismatch between intentions and effective actions may occur [4,27,46], so it is especially relevant to independently measure how the behavioral intention and use behavior may influence the intention to recommend the use of EHR portals:

H10(b): Behavioral intention will positively influence intention to recommend EHR portals to others.

H11: Use behavior will positively influence intention to recommend EHR portals to others.

## Methods

### Measurements

All the items were adopted from the studies by Venkatesh et al [26], Wilson and Lankton [25], van de Kar et al [30], Moore and Benbasat [36], and Oliveira et al [33], with minor changes to adapt to EHR portal technology. The items are exhibited in Multimedia Appendix 1. The questionnaire was delivered in Portuguese after being translated by a certified translator. To guarantee that the content did not lose its original meaning, a back-translation was made from Portuguese to English by a different certified translator and compared with the original [49]. The scales’ items were measured on a 7-point range scale, ranging from “strongly disagree” (1) to “strongly agree” (7). Use behavior was measured on a different scale. The scale from UTAUT2—from “never” to “many times per day”—was adapted to “never” to “every time I need,” because EHR portal usage is not expected to be as regular as mobile internet usage. Sociodemographic questions were also included. Age was measured in years, and gender was coded as a dummy variable (0 or 1), with women represented by 0. Having a private health insurance was also coded as a dummy variable (0 or 1), with its absence represented by 0. Information about the level of education of the respondents was also assessed with 3 different layers (university degree, high school education complete, and high school education incomplete).

### Data Collection

A pilot survey was performed to validate the questions and the scale of the survey. From the pilot survey, we had 20 responses. No issues were reported that could question the fact that the questionnaire items were not reliable. However, from the outcome of the pilot survey, there was strong evidence that our nonresponse rate in the main survey could be high (>50%). The data from the pilot survey were not included in the main survey. As one of the goals of our study is to determine the usage prevalence rate of this type of technology, we subdivided our survey into 2 phases. Two-phase sampling designs are frequently used in epidemiological studies, in health care, when a disease is rare, and when the diagnosis of the disease is difficult or expensive [50]. In the first phase, a bigger random sample from the targeted population is screened with less intensive and expensive screening. In the second stage, a random subsample of the individuals is studied more intensively [50]. We used a similar approach; our target population is also infrequent, but in our case, the aim is to handle a potential high nonresponse rate. Specifically, our population of interest is the Portuguese adult population (age≥18 years) who are users of EHR portals. In the first section, we asked the potential respondent if she or he was a Portuguese adult. If the response was positive, we asked if she or he was a user of EHR portals, and only after identifying that she or he was a user, we asked about her or his interest in replying to our main survey. The EHR portal user is a current user of any of the 4 main functionalities that EHR portals can provide in general (management of health information and communication with health providers, medical appointments schedule, check their own EHR, and request for medical prescription renewals) [1,3-7].

**Figure 2:**
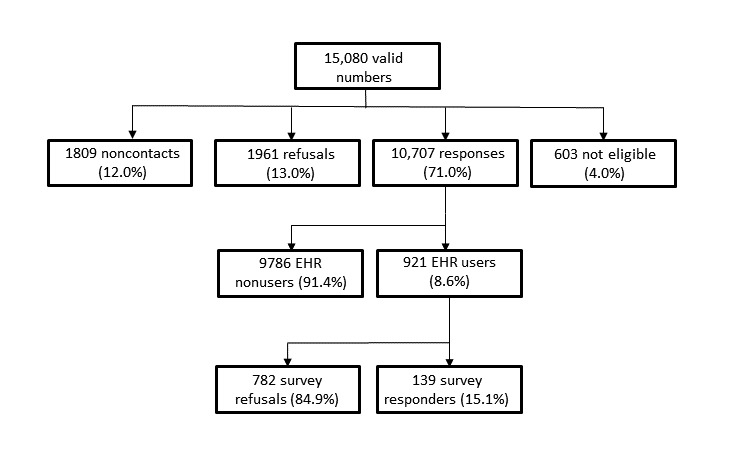
Sampling procedure and results. EHR: electronic health record.

To interview our target population, we used a nationwide mobile phone survey. According to the latest research, 94.5% of the Portuguese adult population had a mobile phone by December 2016 [51], making it a valuable approach to conduct this survey due to its high coverage of the target population. The survey was computer-assisted, and all answers were recorded immediately. The mobile phone sample consisted of randomly generated numbers. Portuguese mobile phone numbers are of 9 digits, and the first 2 digits identify the operator [51,52].

The Portuguese Telecommunications Regulation Authority (ANACOM) delivers information concerning the market share of the 3 operators offering mobile services in Portugal [51]. This was used to split the sample into 3 mobile subsamples proportional to the market share [52,53].

Within each 2-digit prefix of the 3 operators, numbers were created by a generator of 7-digit random numbers [52]. Up to 4 additional call attempts were made to each number to establish contact, with the exceptions when the number was identified as nonworking or not attributed (a message from the operator provides this information) [52,53]. The survey took place between July 25, 2017, and October 15, 2017. All study participants were informed about the research purpose, confidentiality protection, and the anonymity of the information collected and that by answering all the questions, they were giving their consent to participate in the survey. In total, we obtained 15,080 valid numbers. From this sample, we obtained a 71.0% response rate regarding the question to identify the users of EHR portals. From the ones that were eligible to answer the survey, we obtained 139 completed questionnaires and a response rate of 15.1% (see Figure 2).

### Data Analysis

To test our research model, we applied partial least squares structural equation modeling (PLS-SEM). The motivations for choosing this approach were the model complexity (many constructs and many indicators), formatively measured constructs are part of the structural model, and the fact that the PLS-SEM method is oriented to explain variance of the research model and to detect statistically significant constructs [54-56]. SmartPLS 3 [57] was used to estimate the model. Before evaluating the structural model, we assessed the measurement model to evaluate construct reliability, indicator reliability, convergent validity, and discriminant validity.

## Results

### Sample Characteristics

The sample characteristics results versus the target population profile are displayed in Table 2.

Age groups are from 2011census data [58], the level of education uses the latest inquiry from the National Institute of Statistics in 2016 [59] as a source, and for the number of people with private health insurance in Portugal, the information is from the Portuguese Association of Insurance Companies from 2016 [60]. Except for the case of gender, all other sample characteristics differ from the target population. We should not generalize these results as representative of the target population due to the high nonresponse rate in the second phase (Figure 2). Early adopters in eHealth are usually younger and more educated than the general population, in line with the findings of our study [13,38,61]. Higher income is also related to eHealth early adopters, which may justify the higher percentage of people in our sample compared with the target population with private insurance [13,38]. In Portugal, there is a NHS that provides coverage to all citizens, but in the last decade, there was a substantial increase in the number of people obtaining complementary private health insurance [60,62,63]. In Portugal, the main private health care institutions have also implemented measures to encourage the use of eHealth tools, including EHR portals [7].

We also assessed the common method variance initially using Harman one-factor test. If the total variance for a single factor is less than 50%, it suggests that common method variance is not an issue [64]. The greatest variance (47.16%) explained by 1 factor was, in our case by the first one, still lower than 50%. Subsequently, the marker-variable technique was applied, in which we used a theoretical unrelated construct, the marker variable [65]. We found no significant correlation between the research model constructs and the marker variable. Therefore, we can conclude that common method variance was not a serious problem, verified by 2 different and established criteria [64-66].

### Usage Results

According to the results in the first stage of our inquiry, 8.6% of the Portuguese adult population uses EHR portals. This value is within the range of 5%-10%, most commonly reported in the literature [2,14]. We obtained a response rate of 71.0% in the first stage. In the case of our survey, we cannot assume that the nonresponses are *missing at random*, and hence, their lack may lead to a bias [67,68]. According to the literature, the ideal value for responses in a survey should be greater than or equal to 80% to make assumptions about the results and be representative of the population [67,69]. The types of nonresponses in our survey are included in Figure 2. They include 4.0% of individuals who were ineligible, mostly because their age was less than 18 years. Overall, according to other surveys in general and surveys for populations of low prevalence, our response rate may be regarded as reasonable [52,53,70,71].

The usage patterns reported in Table 3 show a good adoption and usage by the users. The feature with the least usage is the request for medical prescription renewals; our sample is relatively young (mean age: 36.0 years). The request for prescription renewals is usually related with chronic conditions that are more prevalent among older people [3,72]. The descriptive statistics of the other questionnaire items are provided in Multimedia Appendix 1.

**Table 2:** Sample characteristics versus target population.

Characteristics		Sample (n=139), n (%)	Population, (n=8,657,240)^a^, n (%)	*P* value^b^
**Age (in years)**				**<.001**
	18-34	67 (48.2)	2,243,957 (25.92)	
	35-49	58 (41.7)	2,367,755 (27.35)	
	50-64	8 (5.8)	2,035,317 (23.51)	
	≥65	6 (4.3)	2,010,211 (23.22)	
**Gender**				**.81**
	Male	64 (46.0)	4,072,366 (47.04)	
	Female	75 (54.0)	4,584,874 (52.96)	
**Private health insurance**				**<.001**
	Yes	78 (56.1)	2,172,967 (25.10)	
	No	61 (43.9)	6,484,273 (74.90)	
**Education**				**<.001**
	University degree	88 (63.3)	1,576,483 (18.21)	
	Nonuniversity degree	51 (36.7)	7,080,757 (81.79)	

^a^Portuguese census 2011 adult population.

^b^*χ*^2^ test.

**Table 3:** Electronic health record portals’ usage patterns.

Use indicators	Average	Median	Minimum	Maximum
UB1: Management of personal information and communication with health providers	4.37	5.00	1.00	7.00
UB2: Medical appointments schedule	4.75	5.00	1.00	7.00
UB3: Check their own electronic health record	4.56	5.00	1.00	7.00
UB4: Request for medical prescription renewals	3.34	3.00	1.00	7.00

### Measurement Model

Typically, the first criterion to be assessed is construct reliability or internal consistency reliability. It is traditionally evaluated by Cronbach alpha, which delivers an estimation of the reliability grounded on the intercorrelations of the observed indicator variables [54]. Cronbach alpha assumes that all indicators are equally reliable. However, PLS-SEM prioritizes the indicators according to their individual reliability [54]. Due to Cronbach alpha’s stated limitations, it is technically more suitable to apply an alternative measure for the same purpose, which is mentioned to as composite reliability [54]. The composite reliability measure takes into account the different indicator variables’ outer loadings [54]. Table 4 shows that all constructs have composite reliability higher than .70, showing evidence of internal consistency [26].

The most commonly used PLS-SEM measure to access convergent validity on the construct level is the average variance extracted (AVE) [54,55]. According to the literature, we should aim to an AVE value of .50 or greater, meaning that on average, the construct explains more than 50% of the variance of its indicators [54,55]. The results in Table 4 demonstrate that this criterion is fully achieved. In addition, to evaluate indicator reliability, a well-known rule of thumb is that a latent variable should explain a significant part of each indicator’s variance, ideally at least half [54,73]. This means that an indicator’s outer loading should be greater than or equal to .70 [54,73]. Nevertheless, indicators with outer loadings between .40 and .70 should be removed only when deleting the indicators leads to an increase in the AVE or the composite reliability above the suggested threshold value [54,55].

Only 1 indicator was removed SP4, with an outer loading below .40. All other indicators have an outer loading higher than .70, and they are shown in Multimedia Appendix 2.

Discriminant validity is the degree to which a construct is truly dissimilar from the other constructs in the model [54]. Traditionally, researchers have relied on 2 measures of discriminant validity [54,55]. One is the Fornell-Larcker criterion that compares the square root of the AVE values with the latent variables’ correlations. Particularly, the square root of each construct’s AVE should be greater than its highest correlation with any other construct [54,55], and as seen in Table 5, this criterion is met. The other traditional measure of discriminant validity is the cross-loadings. Particularly, an indicator’s outer loading on the associated construct should be higher than any of its cross-loadings on other constructs [54,55]. This criterion is also met, as seen in Multimedia Appendix 2. Recent research suggests the use of an alternative criterion, the heterotrait-monotrait ratio (HTMT) of the correlations. HTMT is the ratio of the between-trait correlations to the within-trait correlation [54]. Ideally, the HTMT value should be different from 1; prior research suggests a threshold value of .90 [54]. Ideally, to avoid any ambiguity, the most recent research applied a procedure called bootstrapping to derive a distribution of the HTMT statistic and to determine if it is significantly different from 1 [54]. With this procedure, it is feasible to derive a bootstrap CI (eg, 95%). A CI including the value 1 indicates a lack of discriminant validity. On the contrary, if the value 1 falls outside the interval’s range, this advocates that the 2 constructs are empirically different [54]. This criterion is also met for our model, as seen in Multimedia Appendix 2.

Use behavior, which was modeled using 4 formative indicators, is evaluated by specific quality criteria linked to formative indicators [54]. A recently proposed way to evaluate the formative construct’s validity is to examine its correlation with an alternative measure of the construct, using a global single item or reflective measures (redundancy analysis). The strength of the path coefficients linking the 2 constructs should be at least .70 [54]. In our study, we used a global single item for use behavior, obtaining a path coefficient of .851, thus confirming the convergent validity for the use behavior formatively measured construct. In addition, we need to assess the formative indicators for potential collinearity issues. As seen in Table 6, all variance inflation factors are below 5, meaning that collinearity is not an issue [54]. An additional relevant criterion for evaluating the contribution of a formative indicator is its weight to be statistically significant, or in case it is not significant, its outer loading must be greater than .50 [54]. All formative indicators comply with these assumptions, as shown in Table 6.

**Table 4:** Cronbach alpha, composite reliability, and average variance extracted.

Constructs	Cronbach alpha	Composite reliability	Average variance extracted
Behavior intention	.929	.955	.876
Compatibility	.936	.955	.841
Effort expectancy	.897	.929	.767
Facilitating condition	.822	.883	.655
Habit	.876	.924	.803
Intention to recommend	.879	.942	.891
Performance expectancy	.863	.917	.786
Price value	.953	.970	.915
Results demonstrability	.880	.926	.806
Social influence	.958	.973	.923
Self-perception	.817	.893	.739

**Table 5:** Correlations and square roots of all average variance extracted in the model. Diagonal elements are square roots of all average variance extracted, and off-diagonal elements are correlations.

Constructs	BI	CO	EE	FC	HT	IR	PE	PV	RD	SI	SP	UB
Behavioral intention (BI)	*.936*											
Compatibility (CO)	.809	*.917*										
Effort expectancy (EE)	.561	.645	*.876*									
Facilitating conditions (FC)	.605	.644	.674	*.809*								
Habit (HT)	.703	.616	.541	.534	*.896*							
Intention to recommend (IR)	.826	.779	.610	.593	.585	*.944*						
Performance expectancy (PE)	.695	.651	.481	.468	.537	.648	*.887*					
Price value (PV)	.554	.581	.510	.408	.683	.537	.462	*.956*				
Results demonstrability (RD)	.615	.763	.660	.581	.556	.635	.528	.521	*.898*			
Social influence (SI)	.487	.415	.415	.321	.574	.490	.494	.409	.374	*.961*		
Self-perception (SP)	.514	.432	.224	.333	.552	.401	.494	.243	.449	.380	*.860*	
Use behavior (UB)	.682	.565	.491	.494	.721	.625	.554	.516	.508	.534	.596	formative

**Table 6:** Formative indicators’ quality criteria.

Indicators	Variance inflation factor	*t* value (weights)^a,b^	Outer loadings
UB1: Management of personal information and communication with health providers	1.976	4.923^a^	.892
UB2: Medical appointment schedule	2.432	4.475^a^	.860
UB3: Check their own electronic health record	3.401	0.753	.800
UB4: Request for medical prescription renewals	1.566	1.791	.660

^a^*P*<.01.

^b^*P*<.05.

Considering all the results and findings, all reflective and formative constructs exhibit satisfactory levels of quality. Thus, we can proceed with the evaluation of the structural model.

### Structural Model

Structural model path significance levels were estimated using a bootstrap with 5000 iterations of resampling to obtain the maximum possible consistency in the results [54]. We checked the structural model for collinearity issues by examining the variance inflation factor values of all sets of predictor constructs, and all variance inflation factor values are below the threshold of 5. Therefore, collinearity is not a critical issue in the structural model [54]. To assess the structural model we used the *R*^2^, path coefficients significance, and the *f*^2^ effect size [54,55]. The results are shown in Table 7. Overall, the model explains 76.0% of the variance in behavioral intention and 61.8% in use behavior, with these 2 being the most relevant dependent variables in our model. In addition to assessing the *R*^2^ values of all endogenous constructs, the change in the *R*^2^ value when a specific construct is removed from our model can be used to assess whether the construct has a substantial impact on the endogenous constructs [54]. Guidelines for measuring *f*^2^ are that values of .02, .15, and .35, respectively, represent small, medium, and large effects of the exogenous latent variable; values of less than .02 denote that there is a null effect [54]. Taking a particularly important role in our model, compatibility has a medium effect on both behavior intention and performance expectancy and a small effect on effort expectancy, showing the relevance of this construct in our research model. Another construct with a relevant role in our model is behavior intention, with a large effect on intention to recommend and a small effect on use behavior. Finally, habit is a construct that has a medium effect size on use behavior and a small effect size on behavior intention. With only small effect sizes, we have the effect of performance expectancy on behavior intention, self-perception on use behavior, results demonstrability on effort expectancy, and use behavior on intention to recommend; however, the last one is without a statistically significant path coefficient.

**Table 7:** Structural model results and findings regarding hypotheses.

Dependent and independent variables		*f* ^*2*^	beta	*t_beta_*	Hypothesis	Results	*R* ^2^	*R* ^2^_adj_
**Behavioral intention**							**.760**	**.743**
	Performance expectancy	.081	.203	2.699^a^	H1	Supported		
	Effort expectancy	.001	−.022	.311	H2	Not supported		
	Social influence	.002	.025	.450	H3	Not supported		
	Facilitating conditions	.014	.086	1.547	H4(a)	Not supported		
	Price value	.000	−.015	.277	H5	Not supported		
	Habit	.079	.251	2.660^a^	H6(a)	Supported		
	Self-perception	.008	.062	.916	H7(a)	Not supported		
	Results demonstrability	.015	−.102	1.357	H8(a)	Not supported		
	Compatibility	.328	.530	6.189^a^	H9(a)	Supported		
**Use behavior**							**.618**	**.607**
	Facilitating conditions	.005	.056	.727	H4(b)	Not supported		
	Habit	.165	.378	3.821^a^	H6(b)	Supported		
	Self-perception	.095	.233	2.971^a^	H7(b)	Supported		
	Behavioral intention	.075	.263	2.379^b^	H10(a)	Supported		
**Intention to recommend**							**.690**	**.685**
	Behavioral intention	.962	.747	10.737^a^	H10(b)	Supported		
	Use behavior	.023	.116	1.565	H11	Not supported		
**Effort expectancy**							**.483**	**.476**
	Compatibility	.092	.337	2.243^b^	H9(c)	Supported		
	Results demonstrability	.131	.403	2.888^a^	H8(c)	Supported		
**Performance expectancy**							**.427**	**.418**
	Compatibility	.257	.594	6.141^a^	H9(b)	Supported		
	Results demonstrability	.004	.075	.561	H8(b)	Not supported		

^a^*P*<.01.

^b^*P*<.05.

## Discussion

### Principal Findings

**Figure 3:**
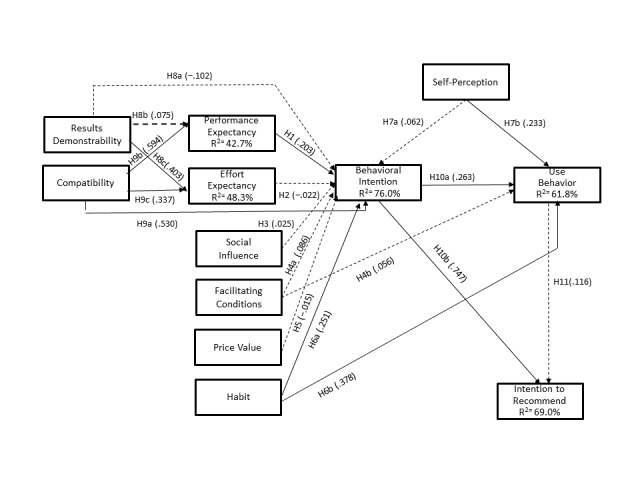
Structural model results. Note: path coefficients that are not statistically significant are in dashed arrows.

The results advocate that using our new research model in an eHealth-related area—EHR portal acceptance by patients—yields very good results, explaining 76.0% of the variance on behavioral intention and 61.8% of the variance in use behavior, the most relevant dependent variables in our model [26]. We also obtained an *R*^2^ of 69.0% in intention to recommend, also a very good result [26,33]. Overall, the use of the 3 theories, UTAUT2, HBM, and DOI, was a successful strategy because in all of them we had constructs with statistically significant impact on explaining the adoption of EHR portals (see Figure 3). The constructs with the highest effect size in the model were compatibility, habit, and behavioral intention.

### Theoretical Implications

In our model, performance expectancy has a statistically significant effect on behavior intention, suggesting that individuals care about the results and advantages that EHR portals can bring for them to manage their own health more effectively, supporting *H1.* This finding is supported by previous studies [25,27]. In regard to effort expectancy, there is no statistically significant impact, not supporting *H2*. This finding contradicts results from earlier studies that used effort expectancy as part of UTAUT2 [2,7], but in other studies also with new technologies and within health care, when effort expectancy is evaluated as part of DOI, it also obtained nonsignificant results [32,33]. A possible explanation, also supported by the literature, is that early adopters of new technologies have a higher cognitive ability and are more used to manage complexity and that they do not perceive it as an obstacle to use EHR portals [32,47].

In our research model, social influence did not show a statistically significant effect on behavioral intention, thus not supporting *H3*. Previous studies have shown potential differences, with results differing among countries, with its positive significance being more consistent in the United States [2,7,27,28]. Potential cultural differences may explain the different behaviors. In our study, our early adopters of EHR portals seem to be more driven by their own individual willingness to try a new technology than to be influenced by what the society generally does. This is also an assumption supported by DOI theory [47]. The nonconfirmation of the facilitating conditions hypothesis, *H4(a)* and *H4(b),* advocates that the individuals in our study believe that the resources or know-how to use EHR portals are not an issue. This can be justified by the ability of having access to a computer and the internet and is aligned with recent literature findings [2,44]. In Europe, access to most of the eHealth services is free of charge; so, the value that is provided to the patients is to permit them to execute specific activities more efficiently online. Unfortunately, that fact seems to be not acknowledged by the patients and *H5* was rejected. Habit has a statistically significant impact on both behavior intention and use behavior, supporting both *H6(a)* and *H6(b)*. Habit is a consumer-specific construct with a very significant role in our model, showing how important it is to have models tailored with consumer-specific constructs and not just general IT adoption constructs [26], and it is also supported by recent literature findings [26,27,44].

Self-perception has a statistically significant impact on use behavior, supporting *H7(b)*, and a nonsignificant impact on behavior intention, not supporting *H7(a)*. Often with sensitive topics and particularly with health-related topics, mismatch between intentions and effective actions occur [4,27,46]. In fact, this is the case with self-perception. Although it does not drive the intentions, self-perception directly influences actions in the usage of EHR portals. Results demonstrability has a statistically significant impact on effort expectancy, supporting *H8(c)*, and a nonsignificant impact on both performance expectancy *H8(b)* and behavior intention *H8(a)*, not supporting these 2 last hypotheses. Our results point out that when an innovation produces results that are readily discernible, perceptions of how easy it is to use a technology are considerably affected (this finding is in accordance with the literature [32]), but not the perceptions related with performance expectancy or a direct influence on behavior intention. Compared with results demonstrability and also from DOI, compatibility has a much greater effect in our research model demonstrated not only by the *f*^*2*^ but also by having all its paths in the model statistically significant. Compatibility has a statistically significant impact on behavior intention *H9(a*), performance expectancy *H9(b)*, and effort expectancy *H9(c)*, supporting these 3 hypotheses. The results indicate that behavior intention *H9(a*), performance expectancy *H9(b)*, and effort expectancy *H9(c)* are greater when the heath care consumer perceives the technology to be compatible. Our study’s results are in line with other studies in this regard [13,32]. Behavioral intention positively influences use behavior, supporting *H10(a*). This finding is in accordance with the literature suggesting that using EHR portals and eHealth tools is preceded by the intention to use them [23,26,27,44]. Behavioral intention also positively influences intention to recommend, supporting *H10(b*). Our model explains 69.0% of the variance in recommendation, and the findings validate the significant influence of behavioral intention over it. Nevertheless, use behavior does not have a significant impact on intention to recommend, not supporting *H11*. A probable explanation might be that being a high user does not necessarily link to higher recommendation, but that a strong intention to use, independent of the usage level, is a stronger predictor of intention to recommend.

### Managerial Implications

The study identifies areas that may influence EHR portal adoption, regarding its conceptualization, implementation, and redesign. Performance expectancy is a significant adoption driver of EHR portals. Therefore, while conceiving and promoting EHR portals, it is relevant to emphasize the advantages that they provide to the users in managing their health-related activities more efficiently. It is also important when conceiving an EHR portal that results are easily demonstrable because perceptions of how easy a technology is to use are affected by them. Compatibility is a very important construct in our model, and it is important to develop EHR portals that fit the health care customers’ lifestyle. A good example is the providers that are already developing mobile versions of their EHR portals, allowing people to access their data everywhere [7]. In addition to the automatic and direct effect of habit on usage, habit also operates as a stored intention path to influence behavior [26]. This requires more communication effort to reinforce both the stored intention and its link to behavior [26]. As habit has been defined as the degree to which individuals tend to execute behaviors automatically due to learning [26], it is advisable that EHR portals have customer support services to help and provide support to the users with the platform.

Another relevant outcome is that the construct that is specific to health care—self-perception—also has a statistically significant role on the EHR portals usage. Self-perception is linked to the fact that the perceived, rather than the real, severity of the health problem is the driving force behind the action [30]. Health care interventions that enable the patient to be more conscious of her or his health condition may also endorse the usage of the EHR portal. In addition, the inclusion of educational health materials in the EHR portals may encourage patients to use the platform. Another important contribution of our study is to be able to demonstrate the influence of the intention to recommend in the adoption of EHR portals. Social network marketing and the opinions shared by friends and relatives are influential ways to help in the promotion and successful adoption of EHR portals. The managerial implications stated here are relevant not only for enhancing the adoption of EHR portals but also for growing the usage frequency of current users. These can be done by developing new EHR portals or by making improvements to existing ones.

### Limitations and Future Research

Unfortunately, our study had a very high nonresponse rate concerning people that refused to answer the main questionnaire. With this high nonresponse rate, it is difficult to make direct assumptions related with the users in the Portuguese population. Nevertheless, earlier literature indicates that users and early users of eHealth tools and EHR portals are younger and more educated than the population average [2,7,13,27,38,61], in line with our study findings. The use of SEM is usually linked with the need of having questionnaires that are not short, making it more difficult for people to answer this questionnaire, especially by phone [52,54,73]. The use of gifts and other incentives may be a useful strategy to overcome the issue of the high nonresponse rate [26]. Testing the research model with samples of EHR users from other countries may also be an interesting path to follow, as the literature has shown that multicountry assessment provides interesting and diverse insights [15,22,27]. We used PLS-SEM instead of covariance based-SEM for the following reasons [54,73]: we have a complex model (many constructs and many indicators), we had the goal of identifying key *driver* constructs, and we also verified that our data were non-normally distributed. We acknowledge that future research may go in the direction of using covariance based-SEM, which allows using global goodness-of-fit criteria, but due to the circumstances and the study goals, we adopted PLS-SEM in our research [54,73].

### Conclusions

Although we acknowledge that we had a very high nonresponse rate in the second stage of our sampling procedure, the much lower nonresponse rate in the first stage provides an estimate of 8.6% usage of these types of platforms in Portugal, a valuable contribution from our study. Our respondents’ demographics follow the same trend as reported in other similar studies in the literature [13,38,61], providing additional support to our findings. Overall, the use of the 3 theories, UTAUT2, HBM, and DOI, to support our research was a successful strategy because in all of them, we had constructs with statistically significant impact on explaining the adoption of EHR portals. We were also able to demonstrate that consumers with a greater intention to adopt a new technology are more likely to become users and to recommend that specific technology to others. The new research model obtained very good results, with relevant *R*^2^ in the most important dependent variables that help to explain the adoption of EHR portals, behavior intention (76.0%), and use behavior (61.8%).

## Appendix

Multimedia Appendix 1 Questionnaire items.

Multimedia Appendix 2 Partial least squares loadings and cross-loadings plus CI for heterotrait-monotrait ratio.

## Abbreviations

AVE: average variance extracted
CFIP: concern for information privacy
DOI: diffusion of innovation
EHR: electronic health record
epSOS: European patients smart open services
HBM: health belief model
HTMT: heterotrait-monotrait ratio
IT: information technology
NHS: National Health Service
PLS-SEM: partial least squares structural equation modeling
TAM: technology acceptance model
UTAUT: unified theory of acceptance and use of technology
UTAUT2: extended unified theory of acceptance and use of technology
