# Supplementary material for: New Integrated Model Approach to Understand the Factors That Drive Electronic Health Record Portal Adoption: Cross-Sectional National Survey
Source: J Med Internet Res. 2018 Nov 19;20(11):e11032. doi: 10.2196/11032 (PMC6318146; doi:10.2196/11032)
Supplement: Multimedia Appendix 1 [file jmir_v20i11e11032_app1.pdf]

Multimedia Appendix 1 Questionnaire's items

Table A1.1- Questionnaire items description

The scales' items were measured on a seven-point Likert scale, ranging from "strongly disagree" (1) to "strongly agree" (7). Use was measured on a different scale (explained in the table below).

**Construct**	**Code**	**Items**	**Reference**
Performance Expectancy	PE1	Using EHR Portals will support critical aspects of my healthcare.	[25]
	PE2	Using EHR Portals will enhance my effectiveness in managing my healthcare.	
	PE3	Overall, EHR Portals will be useful in managing my healthcare.	
Effort Expectancy	EE1	Learning how to use EHR Portals is easy for me.	[26]
	EE2	My interaction with EHR Portals is clear and understandable.	
	EE3	I find EHR Portals easy to use.	
	EE4	It is easy for me to become skilful at using EHR Portals.	
Social Influence	SI1	People who are important to me think that I should use EHR Portals.	[26]
	SI2	People who influence my behaviour think that I should use EHR Portals.	
	SI3	People whose opinions that I value prefer that I use EHR Portals.	
Facilitating Conditions	FC1	I have the resources necessary to use EHR Portals.	[26]
	FC2	I have the knowledge necessary to use EHR Portals.	
	FC3	EHR Portals is compatible with other technologies I use.	
	FC4	I can get help from others when I have difficulties using EHR Portals.	
Price Value	PV1	EHR Portals is reasonably priced.	[26]
	PV2	EHR Portals is a good value for the money.	
	PV3	At the current price, EHR Portals provides a good value.	
Habit	HT1	The use of EHR Portals has become a habit for me.	[26]
	HT2	I am addicted to using EHR Portals.	
	HT3	I must use EHR Portals.	
Self-Perception	SP1	Do you think your health complaints are serious?	[30]
	SP2	Do you think your health complaints have to do with a serious disease?	
	SP3	Do you need more information about your health complaints?	
	SP4	Do you think that you could have treated your health complaints yourself (dropped)	
Results Demonstrability	RD1	I would have no difficulty telling others about the results of using a EHR Portal	[36]
	RD2	I believe I could communicate to others the consequence of using a EHR Portal	
	RD3	The results of using a EHR are apparent to me	
Compatibility	CO1	Using a EHR Portal is compatible with all aspects of managing my health	[36]
	CO2	Using a EHR Portal is compatible with my current situation	
	CO3	I think that using a EHR Portal fits well with the way I like to manage my health	
	CO4	Using a EHR Portal fits in my life style	
Behavioural Intention to recommend	IR1	I will recommend to my friends to use EHR Portals service, if it is available	[33]
	IR2	If I have a good experience with EHR Portals I will recommend friends to use the service	
Behavioural Intention	BI1	I intend to use EHR Portals.	[26]
	BI2	I intend to use EHR Portals in the next months.	
	BI3	I plan to use EHR Portals frequently.	

Construct	Code	Items	Reference
Use Behaviour		What is your actual frequency of use of the following EHR Portal services? (i) Never; to (vii) every time I need it.	[26]
	UB1	Management of Personal Information and communication with health providers.	
	UB2	Medical appointments schedule.	
	UB3	Check your own Electronic Health Record.	
	UB4	Request for medical prescription renewals.	

Note: It was also asked what was the actual EHR Portal global frequency of use. (i) Never; to (vii) every time I need it.

Table A1.2- Questionnaire items descriptive statistics

Item	Average	Median	Maximum	Minimum
UB1	4.37	5.00	7.00	1.00
UB2	4.75	5.00	7.00	1.00
UB3	4.56	5.00	7.00	1.00
UB4	3.34	3.00	7.00	1.00
PE1	5.61	6.00	7.00	1.00
PE2	5.71	6.00	7.00	1.00
PE3	5.57	6.00	7.00	1.00
EE1	5.86	6.00	7.00	1.00
EE2	5.56	6.00	7.00	1.00
EE3	5.15	5.00	7.00	1.00
EE4	5.72	6.00	7.00	1.00
SI1	4.32	4.00	7.00	1.00
SI2	4.37	5.00	7.00	1.00
SI3	4.22	4.00	7.00	1.00
FC1	6.24	7.00	7.00	2.00
FC2	5.93	6.00	7.00	1.00
FC3	5.93	6.00	7.00	1.00
FC4	5.26	5.00	7.00	1.00
PV1	4.64	5.00	7.00	1.00
PV2	4.73	5.00	7.00	1.00
PV3	4.68	5.00	7.00	1.00
HT1	4.41	5.00	7.00	1.00
HT2	4.09	4.00	7.00	1.00
HT3	5.13	5.00	7.00	1.00
BI1	5.54	6.00	7.00	1.00
BI2	5.34	5.00	7.00	1.00
BI3	5.62	6.00	7.00	1.00
IR1	5.42	6.00	7.00	1.00
IR2	5.88	6.00	7.00	1.00
SP1	4.63	5.00	7.00	1.00
SP2	4.35	5.00	7.00	1.00
SP3	5.18	6.00	7.00	2.00
RD1	5.57	4.00	7.00	1.00
RD2	5.63	5.00	7.00	1.00
RD3	5.50	6.00	7.00	1.00
CO1	5.54	6.00	7.00	1.00
CO2	5.63	6.00	7.00	1.00
CO3	5.61	6.00	7.00	1.00
CO4	5.59	6.00	7.00	1.00
