# Supplementary material for: New Integrated Model Approach to Understand the Factors That Drive Electronic Health Record Portal Adoption: Cross-Sectional National Survey
Source: J Med Internet Res. 2018 Nov 19;20(11):e11032. doi: 10.2196/11032 (PMC6318146; doi:10.2196/11032)
Supplement: Multimedia Appendix 2 [file jmir_v20i11e11032_app2.pdf]

Multimedia Appendix 2

Table A2.1 Cross- Loadings

Construct	Item	BI	CO	EE	FC	HT	IR	PE	PV	RD	SI	SP
Behavioural intention (BI)	BI1	**.93**	.72	.50	.56	.62	.75	.65	.47	.55	.43	.45
	BI2	**.93**	.72	.53	.51	.70	.76	.66	.54	.58	.51	.58
	BI3	**.95**	.82	.55	.64	.65	.81	.64	.55	.60	.43	.42
Compatibility (CO)	CO1	.66	**.93**	.61	.58	.54	.69	.52	.53	.72	.38	.38
	CO2	.72	**.95**	.63	.61	.58	.72	.56	.52	.71	.39	.40
	CO3	.80	**.95**	.59	.59	.61	.78	.69	.56	.74	.40	.46
	CO4	.78	**.84**	.54	.58	.52	.66	.61	.51	.63	.36	.35
Effort expectancy (EE)	EE1	.51	.59	**.92**	.69	.42	.53	.42	.36	.61	.28	.14
	EE2	.52	.59	**.93**	.61	.55	.58	.48	.53	.60	.42	.21
	EE3	.44	.50	**.79**	.42	.54	.47	.43	.57	.49	.49	.24
	EE4	.50	.57	**.85**	.62	.40	.56	.36	.36	.60	.29	.20
Facilitating conditions (FC)	FC1	.55	.55	.42	**.78**	.32	.45	.41	.20	.37	.20	.28
	FC2	.46	.50	.70	**.83**	.50	.44	.25	.40	.48	.21	.13
	FC3	.55	.63	.61	**.90**	.48	.59	.51	.38	.58	.29	.40
	FC4	.40	.40	.44	**.72**	.43	.43	.32	.33	.44	.35	.26
Habit (HT)	HT1	.60	.55	.46	.49	**.93**	.47	.46	.62	.46	.49	.50
	HT2	.57	.46	.44	.43	**.92**	.43	.44	.57	.44	.54	.57
	HT3	.72	.65	.55	.51	**.84**	.68	.55	.64	.59	.51	.41
Intention to recommend (IR)	IR1	.85	.76	.57	.56	.66	**.96**	.65	.55	.61	.53	.45
	IR2	.70	.70	.59	.56	.42	**.93**	.57	.45	.59	.38	.29
Performance expectancy (PE)	PE1	.61	.57	.41	.40	.46	.58	**.88**	.32	.51	.42	.50
	PE2	.62	.62	.42	.41	.46	.60	**.93**	.42	.47	.47	.40
	PE3	.62	.54	.45	.44	.52	.55	**.85**	.49	.43	.42	.42
Price value (PV)	PV1	.52	.53	.48	.39	.63	.49	.39	**.93**	.49	.33	.17
	PV2	.54	.59	.49	.38	.63	.53	.46	**.97**	.52	.42	.24
	PV3	.53	.55	.49	.39	.70	.53	.48	**.96**	.48	.42	.29
Results demonstrability (RD)	RD1	.51	.63	.52	.49	.47	.51	.46	.35	**.88**	.32	.44
	RD2	.53	.67	.56	.53	.48	.60	.47	.42	**.95**	.29	.46
	RD3	.61	.74	.68	.53	.54	.59	.49	.60	**.87**	.39	.32
Social influence (SI)	SI1	.50	.42	.37	.30	.54	.48	.48	.38	.37	**.96**	.36
	SI2	.46	.38	.42	.32	.54	.47	.47	.36	.35	**.98**	.38
	SI3	.45	.40	.41	.30	.58	.46	.47	.44	.37	**.95**	.36
Self - perception (SP)	SP1	.47	.37	.16	.27	.51	.33	.45	.23	.30	.31	**.93**
	SP2	.43	.40	.25	.35	0.52	.39	.44	.21	.41	.42	**.92**
	SP3	.44	.35	.18	.23	0.38	.32	.39	.19	.48	.25	**.71**

Table A2.2- Confidence Intervals for HTMT. Average HTMT values computed from 5000 bootstrap samples (column Sample Mean (M))

[b]	Original Sample (O)	Sample Mean (M)	Bias	2.5%[a]	97.5%[a]
CO -> BI	.863	.862	-.002	.776	.920
EE -> BI	.613	.615	.002	.416	.756
EE -> CO	.703	.702	-.001	.498	.838
FC -> BI	.691	.690	-.001	.550	.800
FC -> CO	.729	.726	-.002	.585	.837
FC -> EE	.775	.770	-.004	.611	.897
HT -> BI	.779	.778	-.000	.639	.867
HT -> CO	.679	.676	-.003	.526	.790
HT -> EE	.616	.613	-.003	.449	.746
HT -> FC	.629	.627	-0.002	.458	.764
IR -> BI	.906	.908	.003	.838	.965
IR -> CO	.854	.854	-.001	.775	.916
IR -> EE	.688	.684	-.004	.520	.816
IR -> FC	.695	.690	-.005	.533	.821
IR -> HT	.653	.651	-.002	.478	.771
PE -> BI	.777	.776	-.002	.647	.877
PE -> CO	.720	.718	-.002	.568	.834
PE -> EE	.550	.554	.004	.292	.771
PE -> FC	.552	.552	.000	.336	.741
PE -> HT	.619	.615	-.004	.426	.771
PE -> IR	.740	.743	.003	.504	.888
PV -> BI	.588	.586	-.001	.469	.690
PV -> CO	.614	.612	-.002	.495	.712
PV -> EE	.559	.556	-.003	.383	.706
PV -> FC	.460	.458	-.002	.277	.636
PV -> HT	.747	.746	-.001	.628	.845
PV -> IR	.582	.581	-.001	.461	.693
PV -> PE	.510	.508	-.002	.340	.654
RD -> BI	.674	.674	.000	.540	.791
RD -> CO	.835	.834	-.001	.713	.925
RD -> EE	.733	.726	-.007	.581	.846
RD -> FC	.678	.673	-.004	.502	.812
RD -> HT	.629	.628	-.001	.475	.751
RD -> IR	.718	.714	-.004	.564	.840
RD -> PE	.604	.605	.000	.372	.773
RD -> PV	.558	.556	-.002	.415	.669
SI -> BI	.515	.514	-.001	.351	.648
SI -> CO	.437	.435	-.002	.261	.597
SI -> EE	.456	.453	-.003	.268	.618
SI -> FC	.366	.364	-.002	.193	.534
SI -> HT	.628	.627	-.001	.480	.745
SI -> IR	.525	.525	-.001	.358	.663
SI -> PE	.542	.542	.000	.366	.681
SI -> PV	.428	.426	-.002	.233	.595
SI -> RD	.403	.400	-.003	.225	.556
SP -> BI	.596	.599	.002	.454	.724
SP -> CO	.496	.498	.002	.326	.638
SP -> EE	.266	.276	.010	.102	.467
SP -> FC	.402	.410	.008	.242	.578
SP -> HT	.650	.652	.002	.498	.776
SP -> IR	.467	.470	.003	.306	.608
SP -> PE	.593	.595	.002	.415	.747
SP -> PV	.276	.281	.004	.096	.462
SP -> RD	.552	.553	.001	.416	.676
SP -> SI	.427	.430	.003	.257	.583

Notes:

1. [a] Neither of the confidence intervals includes the value of 1;
2. [b] BI: Behavioural intention; CO: Compatibility; EE: Effort expectancy; FC: Facilitating conditions; HT: Habit; IR: Intention to recommend; PE: Performance expectancy; PV: Price value; RD: Results demonstrability; SI: Social influence; SP: Self-Perception;
